# Stroke in the 21^st^ Century: A Snapshot of the Burden, Epidemiology, and Quality of Life

**DOI:** 10.1155/2018/3238165

**Published:** 2018-11-27

**Authors:** Eric S. Donkor

**Affiliations:** School of Biomedical and Allied Health Sciences, College of Health Sciences, University of Ghana, Accra, Ghana

## Abstract

Stroke is ranked as the second leading cause of death worldwide with an annual mortality rate of about 5.5 million. Not only does the burden of stroke lie in the high mortality but the high morbidity also results in up to 50% of survivors being chronically disabled. Thus stroke is a disease of immense public health importance with serious economic and social consequences. The public health burden of stroke is set to rise over future decades because of demographic transitions of populations, particularly in developing countries. This paper provides an overview of stroke in the 21^st^ century from a public health perspective.

## 1. Introduction

According to the definition proposed by the World Health Organization in 1970, “stroke is rapidly developing clinical signs of focal (or global) disturbance of cerebral function, with symptoms lasting 24 hours or longer, or leading to death, with no apparent cause other than of vascular origin” [1]. Quite recently, a new definition of stroke that incorporates clinical and tissue criteria has been proposed by the American Stroke Association for the 21^st^ century. This definition is much broader and includes any objective evidence of permanent brain, spinal cord, or retinal cell death attributed to a vascular etiology based on pathological or imaging evidence with or without the presence of clinical symptoms [2]. Stroke has a huge public health burden, which is set to rise over future decades because of demographic transitions of populations, particularly in developing countries [3]. This places stroke high on the agenda of public health issues in the 21^st^ century and is an important area for public health research. This paper provides an overview of stroke in the 21^st^ century from a public health perspective and covers several areas of the disease related to the burden, epidemiology, and quality of life.

## 2. Brief Historical Account of Stroke

Stroke was first noted from 460 to 370 before the Common Era by Hippocrates who recorded that occlusion of the “stout” carotid arteries caused loss of consciousness [4, 5]. At this time, stroke symptoms such as convulsions and paralysis were referred to as apoplexy. In 1658, Johann Jacob Wepfer reported that apoplexy resulted from obstruction of the carotid or vertebral artery or bleeding into the brain [4–6]. Around the same time period, Thomas Willis reported on the anastomotic vessels at the base of the brain in his* Cerebri Anatome* [4]. Between 1682 and 1836, it was possible to relate clinical presentations of stroke to morbid anatomical findings of brain through the work of Giovanni Battista Morgagni, John Cheyne, and other scientists [4]. In 1828, John Abercombie clinically classified apoplexy into three groups including primary apoplexy (large intracerebral haemorrhages or infarcts with focal deficits and stupor), probable subarachnoid haemorrhage (with stupor and headache but no focal deficit), and small infarcts or haemorrhages (with focal deficit but no stupor or headache) [4–6]. In the last half of the 20^th^ century, technological revolution produced major advances in antemortem visualization of vascular lesions and discovery of new medical therapeutic strategies for stroke. Angiography invented by Moniz and Seldinger provided valuable information about vascular anatomy. Similarly, Computerized Tomographic (CT) scan and Magnetic Resonance Imaging invented in the 1970s by Hounsfield and Damadian, respectively, have allowed definition of the site of brain infarction and haemorrhage [4]. Newer and more powerful techniques such as Positron and Single-Photon Emission Computerized Tomography are functional imaging procedures that allow the evaluation of cerebral perfusion and metabolism and have provided insights into stroke pathophysiology and discovery of misery perfusion syndrome, diaschisis, and luxury perfusion [4]. These advances in the area of stroke have facilitated the establishment of stroke units that offer effective care for stroke patients and survivors in the 21^st^ century.

## 3. Epidemiology of Stroke

### 3.1. Types of Stroke

Generally, strokes can be classified into two major categories, namely, ischaemic stroke and haemorrhagic stroke. Ischaemic stroke is caused by interruption of the blood supply to a part of the brain resulting in sudden loss of function, while haemorrhagic stroke is attributed to rupture of a blood vessel or an abnormal vascular structure [7]. Generally, ischaemic strokes account for about 80% of stroke cases while haemorrhagic stroke accounts for 20% but the actual proportions of stroke types depend on the population [7]. Data from the first INTERSTROKE study involving 22 countries showed that the proportions of ischaemic and haemorrhagic stroke in Africa were about 66% and 34%, respectively, compared to about 91% of ischaemic stroke and 9% of haemorrhagic stroke in high-income countries [8]. Recent data from the Stroke Investigative Research and Educational Network (SIREN) study in Nigeria and Ghana reported 68% of ischaemic stroke and 32% of haemorrhagic stroke [9], which partly confirms the proportions of stroke subtypes in Africa reported by the INTERSTROKE study. In some countries such as Ghana, there appears to be an evolution of stroke subtypes showing a sharp decline in haemorrhagic stroke and a trend of increasing ischaemic stroke [10–12]. Studies carried out in 1954 and 1981 in Ghana showed that haemorrhagic stroke was the predominant stroke subtype and accounted for approximately 90% of stroke deaths [10, 11]. However, a study carried out between 1994 and 1998 showed that the proportion of haemorrhagic strokes had declined to 60% and cerebral infarction was becoming more common in the country [12].

There are several subclassification schemes for ischaemic stroke and the Trial of ORG 10172 in Acute Stroke Treatment (TOAST) criteria is the most widely used. Based on the TOAST criteria, ischaemic stroke can be grouped into five main pathological or etiological types (Table 1).

There are two types of haemorrhagic stroke including intracerebral haemorrhage and subarachnoid haemorrhage. Intracerebral haemorrhage is the most common type of nontraumatic intracranial haemorrhage; it accounts for 80% of haemorrhagic stroke and 10-15% of all strokes [14]. Intracerebral haemorrhage is mostly caused by uncontrolled hypertension leading to rupture of small vessels. The rupture leads to an avalanche type effect with breakage of nearby vessels resulting in haematoma expansion in up to 40% of cases [4, 14]. Subarachnoid haemorrhage is mainly due to saccular aneurysms though it is also associated with arteriovenous malformation, intracranial neoplasm, and some medications such as anticoagulants [4, 14]. About 65% of subarachnoid haemorrhage patients survive, but half remain disabled primarily due to severe cognitive deficit [4, 14].

Stroke subtypes are reliably determined using CT imaging or Magnetic Resonance Imaging (MRI). Though CT imaging is more commonly used in stroke diagnosis, MRI gives more accurate information and can earlier than CT imaging distinguish between haemorrhage and thrombus [15]. In most developing countries, CT imaging or MRI facilities are not readily available and affordable.

### 3.2. Risk Factors of Stroke

#### 3.2.1. Traditional Risk Factors

Through epidemiological studies, a wide range of stroke risk factors have been identified and are important for primary and secondary prevention of stroke. The traditional risk factors of stroke can be classified into two, which include risk factors that are modifiable and those that are nonmodifiable. The modifiable risk factors of stroke include factors such as hypertension, diabetes mellitus, high blood cholesterol, cardiovascular diseases, sedentary lifestyle, atrial fibrillation, smoking, and alcohol consumption [8, 16]. The nonmodifiable risk factors are relatively few and include factors such as age and gender [8, 16].

Age is the strongest determinant of stroke and the risk of stroke doubles every decade above age 55 [4, 17]. Age can be considered a marker for duration of exposure to other risk factors of stroke [4, 17]. While in Sub-Saharan Africa most stroke cases occur in people less than 60 years, in developed countries stroke usually affects much older people of 70-75 years [18, 19]. Hypertension is the strongest risk factor after age and people with hypertension are about 3 or 4 times more likely to have a stroke [20]. The strong association between hypertension and stroke has been attributed to the powerful effects of hypertension on the cerebral circulation [21]. In cerebral blood vessels, hypertension is known to produce wall hypertrophy and causes reduction in the external lumen diameter of the vessels. In addition, hypertension alters the ability of endothelial cells to release vasoactive factors and increases the constrictor tone of systemic and cerebral arteries [21]. Sickle cell disease, which is relatively more prevalent in Sub-Saharan Africa, is known to increase the risk of stroke by as high as 200-400-fold [22, 23]. Increased haemolysis and changes in rheological properties of Red Blood Cells may be the main factors responsible for the increased risk of stroke among sickle cell patients [22, 23]. A previous stroke significantly elevates the risk of subsequent stroke with a recurrence rate of 5-25% in 1 year and 20-40% in 5 years [4].

Data from the recent INTERSTROKE study indicates that a list of ten stroke risk factors including hypertension, high cholesterol, current smoking, alcohol consumption, diabetes, stress, obesity, heart disease lack of physical activity, and poor diet was responsible for 90% of all strokes [24]. These risk factors, which are predominantly traditional risk factors of stroke, are modifiable, making stroke highly preventable. Several studies have shown that there are variations in the stroke risk factors among different races and populations [18, 25, 26]. In a comparative study of stroke risk factors among stroke survivors in Nigeria and Germany, it was observed that smoking, hyperlipidemia, atrial fibrillation, congestive cardiac failure, ischaemic heart disease, and atherosclerosis of the carotid and vertebral arteries were significantly more common among the German stroke survivors [18]. On the other hand, hypertension was significantly more common among the Nigerian stroke survivors [18]. A biracial study in the UK also showed that cardioembolic and large vessel atherosclerotic stroke were more common in white populations than black populations [26]. The racial differences in stroke risk factors have been attributed to interacting genetic, environmental, dietary, and socioeconomic variables and have implications for the distribution of stroke subtypes [25–27]. While haemorrhagic stroke appears to be more associated with hypertension, ischaemic stroke is more related to factors such as smoking, hyperlipidemia, cardiac disease, and atherosclerosis [28–31]. The relatively common occurrence of the latter group of risk factors in white populations accounts for the predominance of ischaemic stroke in the western world [18, 25, 26]. Western lifestyle is now commonly adopted in many African countries, and thus it is expected that the distribution of stroke risk factors and subtypes in Africa would become similar to what is observed in the western world.

#### 3.2.2. Genetic Risk Factors

Conventional risk factors such as hypertension fail to explain all stroke risk, and there is increasing evidence for the potential pathophysiological role of genes in stroke [32, 33]. Genome wide association studies have identified a wide range of genes associated with stroke [34–39]. Some of these genes include Apolipoprotein E (APOE), methylenetetrahydrofolate reductase (MTHFR), Endothelial Nitric Oxide Synthase (ENOS), Factor V Leiden (F5), Cytochrome P450 4F2 (CYP4F2), beta-fibrinogen, and Phosphodiesterase 4D (PDE4D). Interestingly some of the stroke related genes identified are also associated with the conventional risk factors of stroke. For example, a genome wide association study on an Icelandic population found that risk variants for atrial fibrillation of ch4q25 were also risk factors for ischaemic stroke [35]. The responsible gene is thought to be the* PITX2* gene, which encodes a *β*-catenin-regulated transcription factor [35]. In the Icelandic study, genotyping was done on 1,661 cases of ischaemic stroke and 10,815 control subjects and the most important signals were replicated in two distinct European populations with 2224 cases and 2583 controls [35, 36]. The strongest association occurred with SNP (single nucleotide polymorphism) rs2200733, which showed a strong association with cardioembolic stroke [35]. Additionally, a ch9 variant related to myocardial infarction and coronary artery disease was associated with ischaemic stroke in the different populations [36]. So far, the largest and most recent genome wide association study carried out to identify genes related to stroke is the MEGASTROKE study which involved meta-analysis in 521,612 individuals (67,162 cases and 454,450 controls) sampled from Europe, North and South America, Asia, Africa, and Australia [39]. The investigators identified 32 new stroke risk loci, including 22 novel ones [39]. Like previous genome wide association studies on stroke, the stroke risk loci shared genetic variation with related vascular traits, including blood pressure, cardiac traits, and venous thromboembolism [39].

#### 3.2.3. Other Risk Factors

Several other risk factors of stroke have been proposed but are less documented. Some of these risk factors include oral contraceptive use, vasculitides, inflammatory processes, migraine, sleep apnea syndrome, prothrombin activator inhibitor complex deficiency, hypotension, high C-reactive protein, neurocysticercosis,* Chlamydia pneumoniae*,* Helicobacter pylori*,* Legionella pneumophilia*, chronic bronchitis, periodontal disease, and hyperuricemia [4, 14, 40, 41].

### 3.3. Public Health Burden of Stroke

**Figure 1:**
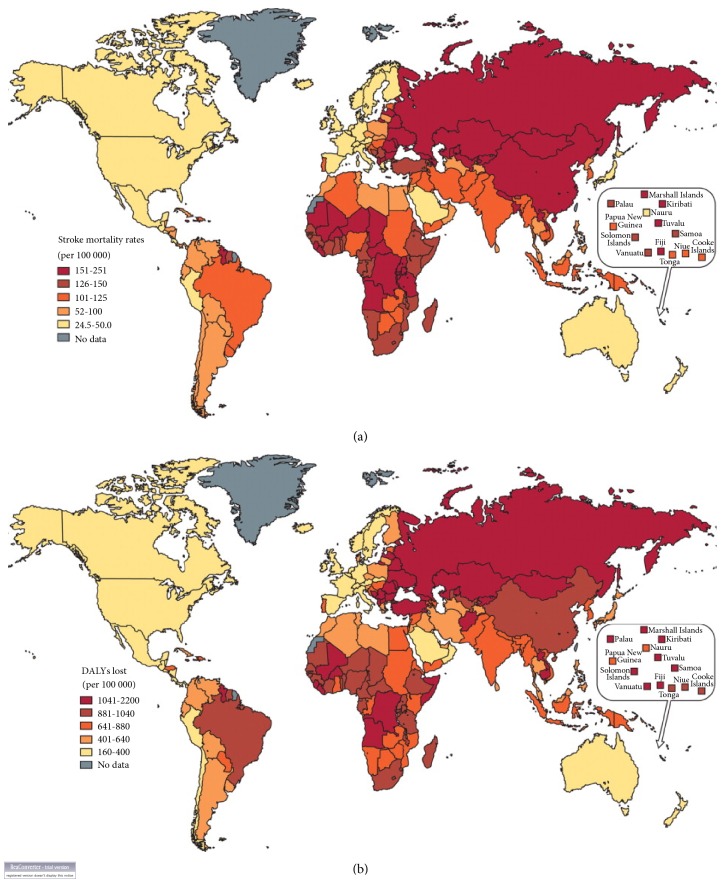
(a) Global distribution of stroke mortality rates. (b) Global distribution of DALY loss due to stroke (source of Figures 1(a) and 1(b): Johnston* et al*. [17]).

Stroke is ranked as the second leading cause of death worldwide with an annual mortality rate of about 5.5 million [16]. Not only does the burden of stroke lie in the high mortality but the high morbidity also results in up to 50% of survivors being chronically disabled [1, 4, 16]. According to the current global burden of disease data on stroke, in 2013 there were almost 25.7 million stroke survivors, 6.5 million deaths, 113 million DALYs due to stroke, and 10.3 million new strokes [42]. Thus stroke is a disease of immense public health importance with serious economic and social consequences. In the past, stroke was considered a disease of the developed world. However, through the application of evidence based control measures, the burden of stroke reduced drastically in many developed countries. In most western European countries, death from stroke declined by 30-50% from 1975 to around 2005 and this was most noticeable in countries like Iceland, Italy, Austria, and Germany [16]. The burden of stroke seems to be shifting to the developing world where currently, there are 4.85 million stroke deaths and 91.4 million DALYs annually compared with 1.6 million deaths and 21.5 million DALYs in high-income countries [42]. As shown in Figure 1, the burden of stroke is much higher in eastern Europe, north Asia, central Africa, and the south Pacific (Figure 1) [17]. In the next few decades, the burden of stroke in the developing world is likely to increase substantially which is partly due to ongoing demographic changes, including ageing of the population and health transitions in these countries [42–45].

The high burden of stroke is partly due to poor community knowledge of stroke risk factors and its warning signs. This is supported by the evidence that increased awareness of stroke risk factors leads to improved compliance with stroke prevention practices while lack of recognition of stroke warning signs is an important causal factor of delay in hospital reporting of stroke [46, 47]. Evidence from studies in developed and developing countries shows that respondents' recognition of any of the established stroke risk factors or warning signs is generally less than 50% [48–53]. In the developing world where the brunt of stroke is currently borne, a study in Benin showed that the most commonly identified stroke risk factor was hypertension (34.5%) while the most often cited warning signs of stroke were paralysis and hemiplegia (34.4%). Similar poor recognition of stroke risk factors and warning signs has been reported in Nigeria and Ghana [49, 50, 54], though one Nigerian study showed good knowledge of stroke risk factors among university staff with 91.7% recognizing hypertension [55].

### 3.4. Pathophysiology of Stroke

The brain, which is the main organ affected by stroke, is metabolically active and needs about 50ml/100g/min blood flow with an oxygen metabolic rate of 3.5cc/100g/min [4, 56]. If the blood flow drops below 10ml/100g/min, brain cell functions are severely affected, while neurons are unable to survive long at levels below 5ml/100g/min [4, 56]. Generally, there is some alteration in brain metabolism if blood flow is interrupted for 30 seconds [4]. In ischaemic stroke, disruption of blood flows to the brain for a few minutes causes hypoxia and hypoglycemia, which leads to infarction of brain tissues [4, 56, 57]. A vicious cycle (ischaemic cascade) ensues due to accumulation of sodium, calcium, and water in the injured brain cells, which lead to release of excitatory neurotransmitters causing further cell injury [56, 57]. In haemorrhagic stroke, the haematoma causes compression of tissue resulting in tissue injury [58, 59]. The brain's regulatory mechanism attempts to maintain equilibrium by increasing blood pressure but the increased intracranial pressure forces out cerebrospinal fluid causing damage to circulation [58, 59]. The blood from brain haemorrhage exerts some direct toxic effects on brain tissue and vasculature [58]. Mass effect ensues with neuronal damage resulting from excitotoxicity, free radicals, apoptosis, ischaemia, diaschisis, neuropathic products, and pressure necrosis [4, 59].

## 4. Stroke and Health Related Quality of Life

In 1948, the World Health Organization defined health as “a state of complete physical, mental and social wellbeing and not merely the absence of disease or infirmity” [60]. Later in 1984, the WHO indicated in a revised statement that any health measure must take into consideration “the extent to which an individual or a group is able to realize aspirations and satisfy needs and to change or cope with the environment” [61]. In connection with this, the WHO quality of life group in 1993 defined “quality of life” as “an individual's perception of his/her position in life in the context of the culture and value systems in which he/she lives, and in relation to his/her goals, expectations, standards and concerns” [62]. The concept of Health Related Quality of Life (HRQoL) is used as an important parameter for measuring outcome in modern medicine and is highly important in the assessment of the multifaceted impact of disease on the patient's life and evaluation of the utility and disability associated with various health states [63, 64]. HRQoL measures encompass emotional, physical, social, and subjective feelings of well-being and hence can be used in identifying and prioritizing areas of need of individual patients and patients with special needs [65, 66]. HRQoL measures are also useful in the evaluation of the effectiveness and cost-benefit of various old and emerging prophylactic, therapeutic, and rehabilitative interventions [64–66]. These instruments facilitate patient caregiver communication and clinical decision-making and uncover hidden problems.

As a result of new therapies, the number of people who survive stroke and live with its consequences is increasing especially in the western world. In the United States, about 85% of stroke victims now survive and currently, there are approximately four million people in the United States alone, who live with the sequelae of stroke [67, 68]. The improvement in survival of stroke patients has necessitated the measurement of the health outcomes associated with stroke prevention, treatment, and rehabilitation. Evidence from stroke studies that have applied HRQoL indicates that outcome measures are important in the identification of determinants of good and poor prognosis in stroke patients [63, 65]. In a study of stroke patients in Auckland, HRQoL was observed to be relatively good for stroke patients compared to normal individuals, and despite significant ongoing physical disability, stroke patients appeared to adjust well psychologically to their illness [69]. On the contrary, a Canadian study showed multidimensional impairment of all HRQoL domains with the exception of the autonomy and purpose of life dimensions [70]. This is supported by the Kansas City study, which showed that even in stroke survivors considered to have recovered, stroke still affected hand function as well as activities of daily living and participation [71]. In a Nigerian study, stroke exerted a multidimensional impact on HRQoL, which was most pronounced in the physical, psychological, cognitive, and social interaction domains [64, 72]. Similar findings have been reported in Ghana with the most affected HRQoL domains being the physical, psychoemotional, and cognitive domains [73]. Recently, van Mierlo* et al*. [74] identified four distinct trajectories for both physical and psychosocial HRQoL: high, low, recovery, and decline. In this study, the investigators identified psychological factors, in particular helplessness and passive coping, as the main predictors of unfavourable HRQoL trajectories. Evidence from various studies indicates that determinants of HRQoL of stroke patients cover a wide spectrum including depression, social support, caregiver characteristics, social class, functional status, age, comorbidity, laughter frequency, and time elapsed after stroke [64, 69–76].

Measures of HRQoL could be generic or disease-specific [63]. Generic measures assess and compare HRQoL across populations or different diseases, while disease-specific measures are more valid, patient-centered, responsive, and sensitive in assessing HRQoL in specific diseases and/or populations [77, 78]. Examples of generic HRQoL measures are SF-36 and EuroQol [78, 79]. Stroke-specific measures include the Niemi QoL scale Stroke Impact Scale (SIS), Stroke and Aphasia Quality of Life Scale -39 (SAQoL-39), Newcastle Stroke-Specific Quality of Life Measure (NEWSQOL), Stroke-Specific Quality of Life Scale (SSQoL), and the Health Related Quality of Life in Stroke Patients (HRQoLISP) [80–84].

Disability has a profound effect on HRQoL in stroke [64, 70–76, 85]. The International Classification of Impairments, Disabilities and Handicaps (ICIDH) defined disability as any restriction or lack (resulting from an impairment) of ability to perform an activity within the range considered normal for a human being [82]. Disability measures include modified Rankin Scale, Barthel Index, AHA Stroke Outcome Scale, and the Stroke Levity Scale [86, 87]. These instruments assess basic and daily functioning such as feeding, dressing, bathing, toileting, and mobility. The major challenge of activities of daily living (ADL) measures is that no instrument can adequately represent the numerous ADL factors in each person and consequently a single case design has been proposed [88].

## 5. Stroke and Infections

Neurological impairment in stroke is known to cause immunodepression, and consequently stroke patients are relatively more susceptible to infections [89]. A systematic review of 87 studies involving 137817 inpatients reported an overall pooled infection rate of 30%; rates of pneumonia and urinary tract infection were 10% each [90]. It is important to note that the data included in this systematic review was largely from the developed world and there was hardly any data from Sub-Saharan Africa. In Ghana, a recent study reported a significantly higher prevalence of UTI among stroke outpatients (24.3%) than among a healthy control group (7.2%) [91], while another study reported a significantly higher prevalence among stroke inpatients (18.8%) than among outpatients (10.9%) [92]. Risk factors of UTI among stroke patients include stroke severity, depressed conscious level, increased post-void residual urine volume, catheterization, and diabetes mellitus [90]. A number of bacterial pathogens have been implicated in UTI among stroke patients and the major ones include* Staphylococcus aureus* and gram-negative bacteria such as* Pseudomonas aeruginosa*,* Escherichia coli*, and* Enterobacter* spp. [90–92]. Compared to UTI, pneumonia is a more serious complication following stroke and is commonly caused by* Klebsiella pneumoniae*,* Pseudomonas aeruginosa*, and* Escherichia coli* [90, 93]. Stroke related pneumonia tends to be associated with aspiration and its related risk factors such as impaired level of consciousness and dysphagia [93]. It is thought that prophylactic antibiotics may offer some benefit against UTI and pneumonia in stroke patients. However, the use of such drugs after stroke is still unclear, and questions concerning the risk of selecting resistant strains, defining the best antibiotic regimen, and determining which patients with stroke may benefit from prophylaxis remain unresolved [73, 94].

## 6. Control of Stroke

Prevention of occurrence of new strokes is the key solution to the problem of the growing stroke burden. The WHO advocates a combination of population-wide and high-risk approaches to prevent stroke and other types of cardiovascular diseases (CVD) [95]. The high-risk prevention strategy involves determination of absolute risk of CVD over the next 5 or 10 years to help identify people at high risk of development of acute CVD. The population-wide prevention strategy targets several behavioural and lifestyle risk factors and this approach is essential, because even small favourable changes in the distribution of risk factors could lead to major reductions in stroke and CVD incidence in the population. Some other primary prevention strategies of stroke include community-based education programmes and digital health technology [96]. Another important consideration in the control of stroke is the prevention of secondary vascular events. For both ischaemic and haemorrhagic stroke, appropriate control of blood pressure reduces the risk of subsequent strokes [18, 26]. For patients who have suffered an ischaemic stroke, antiplatelet therapy and cholesterol reduction are also important for secondary prevention [97, 98].

For management of stroke during the acute stage, some surgical procedures and medications may be needed and inpatient stroke care units have been proven to provide high quality care especially in the developed world. Stroke survivors may require lifelong pharmaceutical treatment, rehabilitation, and caregiver support in order to achieve optimal health outcomes. An important component of the long-term treatment of stroke patients is managing sequelae such as swallowing difficulties, depression, and spasticity.

Stroke surveillance forms an important component of the overall control of stroke as it provides information about etiology, risk, prognosis, prevention, and intervention as well as disease distribution and time trends. Two sources of data are available for the purposes of surveillance: primary data, such as those arising from health surveys or local population-based registries, and secondary data, arising from large administrative databases. The WHO has proposed a surveillance system for stroke involving three steps, which represent the possible initial outcomes for stroke patients: events in hospital, fatal events in community, and nonfatal events in community [99]. The WHO stroke surveillance system begins with cases admitted to hospital, as this group is the one most easily identified and follows these patients until discharge or death. The second level of complexity involves identifying and validating the diagnoses for fatal instances of stroke where the patient is not admitted to a hospital, i.e., the fatal events in the community. The third step represents nonfatal nonhospitalised events. The WHO stepwise approach to stroke surveillance provides a flexible system and an opportunity for all countries to contribute data on stroke. The level of complexity will depend on development of health services and resources, and each participating country may collect precisely the amount of data that it finds feasible.

## 7. Conclusion

Stroke remains a disease of immense public health significance in the 21^st^ century despite the advances in our understanding of several important areas of the disease such as the epidemiology, quality of life, and pathophysiology. In both the developed and developing world, ischaemic stroke is currently the predominant stroke subtype. Hypertension remains the leading risk factor of stroke in both developed and developing countries despite the racial differences in the risk factors of stroke. Since the burden of stroke is expected to increase significantly in future, there is the need for a better understanding of the factors associated with high blood pressure, especially in countries with a high risk of stroke. Generally, little is known about HRQoL of stroke survivors globally and the affected HRQoL domains in stroke patients appear to vary geographically or culturally. In the developing world, where there are limited rehabilitation facilities, it is essential to identify and modulate the factors affecting HRQoL of stroke survivors in order to promote maximal HRQoL improvements in these patients. Infections constitute an important complication in stroke patients but have received very little attention in stroke research particularly in the developing world. Understanding infection patterns in stroke patients including the antimicrobial susceptibility patterns of bacterial etiological agents could contribute to improvement in stroke treatment outcomes.

## Figures and Tables

**Table 1:** Types of ischaemic stroke based on the TOAST classification.

**Stroke type**	**Causes**	**Percentage**
Large artery thrombotic strokes	Atherosclerotic plaques in the large blood vessels of the brain lead to ischemia and infarction	20%

Small penetrating artery thrombotic stroke (Lacunar stroke)	One or more vessels in the brain are affected (microatheromatosis)	25%

Cardiogenic embolic stroke	Associated with cardiac dysrhythmias, valvular heart disease, and thrombi in the left ventricles	15%

Cryptogenic strokes	Cause is unknown	5-10%

Strokes associated with other causes	Such as illicit drug use	20-25%

Source: adapted from Adams *et al*. [13].
